# A school-based resilience intervention to decrease tobacco, alcohol and marijuana use in high school students

**DOI:** 10.1186/1471-2458-11-722

**Published:** 2011-09-24

**Authors:** Rebecca K Hodder, Justine Daly, Megan Freund, Jenny Bowman, Trevor Hazell, John Wiggers

**Affiliations:** 1 Hunter New England Population Health, Hunter New England Area Health Service, New South Wales, Australia; 2 The University of Newcastle, New South Wales, Australia; 3 Hunter Medical Research Institute, New South Wales, Australia; 4 Newcastle Institute of Public Health, New South Wales, Australia; 5 Hunter Institute of Mental Health, Hunter New England Area Health Service, New South Wales, Australia

## Abstract

**Background:**

Despite schools theoretically being an ideal setting for accessing adolescents and preventing initiation of substance use, there is limited evidence of effective interventions in this setting. Resilience theory provides one approach to achieving such an outcome through improving adolescent mental well-being and resilience. A study was undertaken to examine the potential effectiveness of such an intervention approach in improving adolescent resilience and protective factor scores; and reducing the prevalence of adolescent tobacco, alcohol and marijuana use in three high schools.

**Methods:**

A non-controlled before and after study was undertaken. Data regarding student resilience and protective factors, and measures of tobacco, alcohol and marijuana use were collected from grade 7 to 10 students at baseline (n = 1449) and one year following a three year intervention (n = 1205).

**Results:**

Significantly higher resilience and protective factors scores, and significantly lower prevalence of substance use were evident at follow up.

**Conclusions:**

The results suggest that the intervention has the potential to increase resilience and protective factors, and to decrease the use of tobacco, alcohol and marijuana by adolescents. Further more rigorous research is required to confirm this potential.

## Background

Tobacco, alcohol and other drug use contribute significantly to mortality and morbidity in many countries [1,2]. Tobacco use generally commences in early adolescence [3], with earlier uptake associated with heavier smoking [4], rapid establishment of nicotine dependence even after brief intermittent use [5] and greater difficulty in quitting in adulthood [4]. Similar to tobacco, initiation of alcohol use generally occurs in adolescence [6], and earlier drinking experiences have been linked to alcohol dependence in adulthood [7]. The patterns of illicit substance misuse developed in youth are similarly associated with continued use into adult life [8]. World wide, a significant proportion of adolescents use tobacco, alcohol and marijuana, with such use being greater in older adolescent age groups [9-13].

Schools are considered an ideal setting for programs aimed at decreasing the prevalence of health risk behaviours as: they provide access to young people at a time when they are vulnerable to emotional problems and risk taking behaviour [14]; young people spend half their waking hours at school; and the quality of experiences with teachers and peers can have a positive impact on young people's health and emotional well-being [15]. Despite such potential, reviews of school-based programs designed to reduce the prevalence of tobacco and alcohol use have found conflicting or little evidence of effect [16-19]. In particular, interventions focused on the provision of information (for example, interventions that only include information-giving curricula [20]) have been suggested to be ineffective [19]. A World Health Organisation review of school health promotion interventions further concluded that programs promoting young people's mental well-being were the most likely to be effective, recommending such an approach be the focus of future studies targeting adolescent substance use [18]. The review also suggested that interventions that incorporate changes in the school curriculum, the school environment and that foster relationships between schools and their communities were the most likely to achieve a beneficial outcome, an approach known as the 'health promoting schools' framework [18]. Such a view is supported by research that identifies school culture to be a determinant of substance use [21,22].

Resilience theory, which has arisen from the study of risk factors and their impact on positive youth development, represents one approach to improving adolescent mental well-being [23-29]. Whilst there is much variation in the definition of resilience, it is generally agreed that both individual as well as environmental characteristics contribute to individual resilience and are critical for positive youth development and the avoidance of risk behaviours [30-33]. Individual characteristics, termed resilience factors, refer to the personal skills and traits of young people, and include self-esteem, empathy, help-seeking and self-awareness [34]. Where as protective factors refer to positive influences within a young person's environment such as family, school, and community connection [34]. As associations between such characteristics and substance use have been reported [35-37], interventions designed to increase such factors may represent a means of reducing the extent of adolescent substance use uptake.

Although a number of school-based trials have targeted resilience or protective factors to reduce substance use [34,38-42], no controlled studies could be identified that described the effectiveness of an intervention that targeted both types of factors using the health promoting schools framework. Of the controlled trials that have incorporated a focus on either resilience or protective factors, inconsistent effects on tobacco, alcohol and marijuana use have been reported [34,38-42]. For example, in Australia, a three year cluster randomized controlled trial involving 26 secondary schools assessed the effect of a social and school connectedness intervention on student tobacco, alcohol and marijuana use [40,40]. One and two year follow up data were collected for a cohort of students recruited in Grade 8. At one year follow up (students in Grade 9), a significantly greater reduction in substance use was only found for smoking [40], whilst at two year follow up (students in Grade 10) no significant effect was found for smoking or alcohol, but a significant reduction in marijuana use was found [39]. On further subgroup examination the authors found a greater intervention effect for marijuana use in Grade 10 if students were nonsmokers in Grade 7 and for those who reported the lowest level of school engagement in Grade 8 and 9 [39]. The authors concluding this type of intervention may only be effective if implemented prior to initiation to tobacco smoking and for those students considering experimentation with marijuana use who are least engaged in school [39]. This conclusion is supported by studies that have demonstrated exposure to intervention prior to target problem uptake is predictive of greater effectiveness [43].

One non-controlled evaluation of an intervention addressing both resilience and protective factors using a health promoting schools approach has been reported [34,41]. The intervention aimed to reduce risk behaviours, including tobacco, alcohol and marijuana use, among students in fifteen non-randomly selected Australian secondary schools. Using a cross sectional design, three year follow up data were obtained from students in Grades 7 to 11 [34,41]. No significant effect on substance use was found. In addition, of 30 post hoc analyses by student grade and gender, significant reductions were found in only six cases: smoking by Grade 7 males and by Grade 8 males and females; alcohol use by Grade 7 males and Grade 8 females; and marijuana use by Grade 9 males.

A separate process evaluation was conducted after the initial non-controlled evaluation to identify factors that may have contributed to these inconsistent results. The authors identified limited uptake of the intervention by schools, in particular, a whole of school approach to intervention adoption was implemented by less than half of schools, only one-third had implemented recommended intervention planning and monitoring mechanisms, and only 20% had developed recommended relationships with external agencies [41]. Interviews with school staff identified a number of barriers to intervention implementation including: inadequate resources; inadequate levels of school staff professional development; inadequate school executive support; and the importance of funding to ensure sustainability [41]. Such barriers are consistent with those suggested by other studies to limit intervention uptake and fidelity, and hence intervention effect [44-46]. These findings, combined with those from other school-based studies which recommend comprehensive and systematic approaches to intervention implementation [29], suggest that future interventions of this type include explicit strategies to address such barriers and foster intervention uptake and fidelity. Despite this, whilst studies addressing student resilience have since reported some adoption strategies [40], at the time of development no studies could be located that reported explicit and comprehensive program adoption strategies.

Given the limited number of studies examining the effect of comprehensive interventions that address both resilience and protective factors on adolescent substance use, and the lack of reported studies that report the use of strategies to support the adoption of such an intervention, the aim of this pilot study was to examine the potential efficacy of a resilience-based intervention supported by adoption strategies on modifying adolescent resilience and the extent of adolescent substance use uptake.

## Methods

### Design

A non-controlled repeat cross sectional study was undertaken. The intervention was implemented over 3 years in each school across Grades 7 to 10. Cross sectional data were collected prior to intervention implementation and again 12 months following completion. The outcome measures of interest were student reported resilience and protective factor scores, and tobacco, alcohol and marijuana use.

### Setting and sample

#### Schools

Three public high schools were selected on a convenience basis. The schools were located within a 15 kilometre radius of each other in one regional area [47] of New South Wales, Australia. The region has a population of approximately 50,000 people, with an estimated 3,600 people aged 12 to 16 [48], and is ranked in the lowest quintile of socio-economic disadvantage using 2006 Australian Bureau of Statistics SEIFA Index of Relative Socio-Economic Disadvantage [49]. Ethics approval was obtained from the New South Wales Department of Health.

#### Students

The data were collected in August 2002 (baseline) and June 2006 (follow-up). At baseline, the schools ranged in size from 593 to 1011 students. All Grade 7-10 students (aged 12-16 years) attending the three high schools were eligible to participate (2002: n = 1899; 2006: n = 1965). Students were blinded to the study aim of decreasing health risk behaviours.

### Procedures

#### Recruitment and consent

An information letter and consent form were provided to each student by the school to obtain parental consent. Non-responding parents were followed up by phone to prompt return of the consent form.

#### Resilience and protective factor intervention

A multi-strategic intervention based upon an existing student resilience and protective factor program was implemented [50]. Data obtained from a baseline survey were used to inform the selection of resilience and protective factor intervention strategies in each of the three health promoting schools domains: curriculum, teaching and learning; ethos and environment; and partnerships and services [18].

##### Curriculum, teaching and learning

Strategies involved the implementation of various curriculum materials and programs designed to enhance student resilience and protective characteristics including curriculum materials designed to enhance student communication, connectedness, empathy and self-awareness across all grade [50]; and implementation of programs targeting particular resilience and protective factors, such as the Rock and Water Program [51] or the Resourceful Adolescent Program [52].

##### Ethos and environment

Strategies involved the development and modification of school policies and programs relating to bullying to increase school connectedness, enhancement of peer support program to increase school connection and self esteem, and student recognition programs to enhance student autonomy, goals and aspirations via acknowledgement of student achievements.

##### Partnerships and services

Strategies involved schools forming formal partnerships with local services to provide youth services access within school hours to enhance help seeking, initiatives to promote greater parent involvement via active engagement in school-initiated activities and promotion of links with community organisations with the school.

#### Strategies to enhance school intervention adoption

To maximise intervention adoption by schools the following strategies were implemented based on evidence of their effectiveness in supporting practice change in human service organisations [53] and findings from other school-based studies [29,41,45,46,54,55]:

##### Local consensus and adaptation

A number of strategies were implemented to ensure appropriate leadership support was available during intervention implementation, and the strategies implemented were feasible and able to be integrated within existing school systems [45]. Strategies implemented at each school included: the development of a memorandum of understanding to outlining the partnership between, and the roles of, schools and researchers [56]; formation of an advisory group to guide the intervention; establishment of core teams to implement the intervention [55]; and intervention planning workshops for school staff, parents and community members [45].

##### School action plan and performance monitoring

A school action plan [54] was developed by each school based on the results of biennial student resilience and protective factors surveys. The surveys further provided a means of monitoring and reviewing the action plan implementation and effectiveness, with schools being provided reports of their student results.

##### Staff training

Core staff from each school participated in annual training programs to increase their capacity to address student resilience, communication, connectedness, empathy and self-awareness [34].

##### Provision of intervention implementation resources

One full time research assistant was employed for three years to support the three schools to implement the intervention. In addition, for the two initial intervention years, funding was provided to each school to facilitate teacher participation in training, planning and implementation of the intervention (AUS$4,000 and AUS$5,000 respectively per school).

### Data collection

Students at each school completed a pen and paper survey conducted within class time at both baseline and follow up data collection. The survey included items addressing student resilience and protective factor characteristics, and their substance use behaviours.

#### Measures

##### Resilience and protective factor scores

The survey, based on the resilience module from the California Healthy Kids Survey [30,34,57], included items relating to six resilience and six protective factor subscales. The six resilience factor subscales included items addressing the following: empathy (2 questions), effective help seeking (3 questions), self esteem (3 questions), communication and cooperation (2 questions), self awareness (2 questions), and goals and aspirations (2 questions). The six protective factor subscales included items regarding: family connection (4 questions), pro-social peers (3 questions), autonomy experience (4 questions), community connection (4 questions), school connection (4 questions), and pro-social group (3 questions). Students responded to each question using a four-point Likert scale ranging from '1 - never true', to '4 - true all of the time'. The subscales have been shown to have excellent to adequate internal reliability (resilience factors a = 0.53-0.78; protective factors a = 0.69-0.89) [34] and to be reliable and valid in an Australian school population [58].

##### Tobacco, alcohol and marijuana use prevalence

Questions regarding student use of tobacco (3 questions), alcohol (2 questions) and marijuana (1 question) were based on items from previous surveys conducted within New South Wales secondary schools (Table 1) [11,34].

**Table 1:** Student health risk behaviour items

Health risk behaviours questions	Responses
**Tobacco**	
Have you ever smoked even part of a cigarette?	No; Yes, just a few puffs; Yes, less than 10 cigarettes in the last 3 months; Yes, between 10 and 100 cigarettes in the last 3 months; Yes, more than 100 cigarettes in the last 3 months. ^a^
In the last 3 months I have smoked one or more cigarettes on:	No days; 1 day; 2 days; 3 days; 4-5 days; 6-10 days; More than 10 days. ^b^
At the present time, do you smoke cigarettes:	Daily; At least once a week; Less than once a week; Not at all.
**Alcohol**	
In the last 3 months I have had one or more drinks of *beer, wine *or *spirits *(do not count sips or tastes) on:	No days; 1 day; 2 days; 3 days; 4-5 days; 6-10 days; More than 10 days. ^b^
In the last four weeks, how many times have you had 5 or more alcoholic drinks in a row?	None; Once; Twice; 3-6 times; 7 or more times.
**Marijuana**	
In the last 3 months I have used marijuana on:	No days; 1 day; 2 days; 3 days; 4-5 days; 6-10 days; More than 10 days. ^b^

^a^New South Wales School Students Health Behaviour Survey [11]

^b^MindMatters Evaluation Project [34]

##### Student characteristics

Students were asked to specify their grade and gender.

### Analysis

#### Sample characteristics

Student descriptive characteristics (gender and grade) at baseline and follow up were compared using Chi square analysis.

#### Resilience and protective factor scores

At baseline and follow up, individual student scores for each of the six resilience and six protective factor subscales were calculated by averaging responses to questions in each subscale. An overall resilience and protective factor score for each student was calculated by summing these subscale scores.

Resilience factor and protective factor scores for each school, and for all three schools combined, were calculated by averaging all individual student scores. As such scores were not normally distributed, median scores are reported, and differences between scores at baseline and follow up were examined using the Fisher Exact Test (non-parametric ANOVA).

#### Prevalence of tobacco, alcohol and marijuana use

Responses to the tobacco, alcohol and marijuana use items were categorised to form six outcome measures: use of tobacco, alcohol, and marijuana in the last three months (any, none); ever smoked a cigarette (yes, no); current smoking (yes, no); and consumption of five or more alcohol drinks in a row in the last four weeks (any, none). Differences between baseline and follow up in the proportion of students reporting each of the six outcomes were examined by Chi square analysis for all three schools combined, for each school separately, and by grade and gender. A significance level of *p *≤ 0.01 was used to adjust for multiple testing for substance use outcomes [59].

All analyses were undertaken using SAS Software Version 8.2 [60].

#### Sample size

Allowing for a potential intra school correlation of 0.01 [61], and a response rate of 50%, a difference in resilience and protective factor scores for the three schools combined of 0.8 was estimated to be detectable based on a sample size of 900 students at the three schools at baseline and follow up (80% power, *p *= 0.05). Using these same parameters [62] and a baseline prevalence of 50%, a 10% difference in student reported tobacco, alcohol and marijuana use was estimated to be detectable.

## Results

### Student sample

At baseline and follow up, 1449 (76.3%) and 1205 (61.3%) students respectively with parental consent participated in the study (Table 2). The proportion of females (*p *= 0.14), and the proportion of students by grade (*p *= 0.32) who participated in follow up data collection were not significantly different to those participating at baseline. The gender and grade characteristics of participating students at both data collection points were similar to students in New South Wales public secondary schools [63].

**Table 2:** Participant descriptors

Participant Descriptors	2002 n (%)	2006 n (%)	*p *value
TOTAL	1449 (76.3)	1205 (61.3)	
School			
A	425 (78.0)	331 (69.0)	*p *= 0.34
B	577 (79.8)	514 (62.8)	
C	447 (70.7)	360 (54.0)	
Gender			
Female	709^c^	626^d^	*p *= 0.13
Male	734	577	
Grade			
7	383^a^	318^b^	*p *= 0.32
8	358	317	
9	335	298	
10	367	271	

^a^6 students did not provide gender

^b^2 students did not provide gender

^c^6 students did not provide grade

^d^1 student did not provide grade

### Intervention delivery

The intervention strategies implemented by schools differed in emphasis according to the priorities identified by each school. The total number of strategies targeting resilience and protective factors over the three year intervention period ranged from 27 to 39 per school (School A: 6-14 per year; School B: 4-12 per year; School C: 2-17 per year). Of the strategies implemented across the three schools, 26-53% addressed the curriculum, teaching and learning domain; 31-56% ethos and environment; and 16-21% partnerships and services.

### Resilience and protective factor scores

The combined median resilience factor score for the three schools at follow up (18.17) was significantly greater compared to that at baseline (18.00) (*p *< 0.01). Similarly, the median protective factor score for the three schools combined at follow up (17.67) was significantly greater than that at baseline (17.25) (*p *< 0.01)) (Table 3). On an individual school basis, at follow up a significantly greater median resilience factor score was evident for School A only (*p *< 0.01), with a trend toward a greater resilience factor score at follow up for School B. Significantly greater median protective factor scores were evident for Schools A (*p *< 0.01) and B (*p *< 0.05) at follow up.

**Table 3:** Overall median resilience and protective factor scores

Overall factor scores	**2002**^**a**^	**2006**^**a**^	*p *value
***Resilience***			
All schools	18.00	18.17	<0.01
School: A	17.83	18.50	<0.01
B	18.00	18.17	0.07
C	17.83	17.83	0.41
***Protective***			
All schools	17.25	17.67	<0.01
School: A	17.17	17.83	0.01
B	17.33	17.75	<0.05
C	17.17	17.17	0.56

### Tobacco, alcohol and marijuana use prevalence

At follow up, the proportion of all students that reported substance use for each of the 6 outcome measures was significantly lower than that at baseline (Table 4). For smoking outcomes, the proportion of students in all three schools combined who reported: ever smoking was 23.8% less (*p *< 0.01); smoking in the last three months was 12.9% less (*p *< 0.01); and being a current smoker was 12.0% less (*p *< 0.01). The proportion of students who reported consumption of one or more alcoholic drinks in the last 3 months was 19.2% less (*p *< 0.01), and consumption of five or more drinks on one or more days was 16.4% less (*p *< 0.01). Student report of marijuana use in the last 3 months was 9.5% less (*p *< 0.01).

**Table 4:** Prevalence of student tobacco, alcohol and marijuana use^a^*

	TOBACCO						ALCOHOL				MARIJUANA	
	**Ever smoked**^**b**^		**Last 3 months**^**c**^		**Current smoker**^**d**^		**Last 3 months**^**e**^		**Binge drinking**^**f**^		**Last 3 months**^**g**^	
	2002 n (%)	2006 n (%)	2002 n (%)	2006 n (%)	2002 n (%)	2006 n (%)	2002 n (%)	2006 n (%)	2002 n (%)	2006 n (%)	2002 n (%)	2006 n (%)
**All students**												
	714 (50.6)	309 (26.8)	352 (24.9)	137 (12.0)	334 (23.2)	135 (11.2)	687 (48.7)	334 (29.5)	483 (33.8)	204 (17.4)	231 (16.3)	77 (6.8)
**School**												
A	212 (51.0)	103 (31.5)	102 (24.9)	48 (14.7)	100 (24.6)	42 (13.0)	194 (48.1)	94 (28.8)	143 (33.8)	61 (18.8)	68 (16.6)	23 (7.1)
B	289 (50.9)	120 (24.6)	143 (25.2)	52 (10.5)	129 (22.9)	54 (11.0)	283 (49.8)	151 (30.8)	198 (34.6)	85 (17.0)	85 (15.0)	25 (5.0)
C	213 (49.9)	86 (25.2)	107 (24.4)	37 (11.6)	105 (24.7)	39 (11.5)	210 (47.8)	89 (28.2)	142 (32.8)	58 (16.6)	78 (17.7)	29 (9.1)
**Grade**												
7	110 (29.2)	31 (10.6)	33 (8.8)	9 (3.1)	43 (11.3)	7 (2.2)	83 (22.1)	31 (10.8)	53 (13.9)	12 (3.9)	18 (4.8)	2 (0.7)
8	173 (50.1)	63 (20.5)	96 (27.3)	25 (8.1)	91 (25.4)	30 (9.5)	158 (45.1)	62 (20.3)	103 (29.3)	33 (10.7)	52 (14.8)	17 (5.6)
9	193 (59.4)	104 (36.2)	97 (30.0)	49 (17.3)	90 (27.0)	50 (16.8)	192 (58.9)	105 (37.6)	134 (40.5)	74 (25.3)	70 (21.4)	28 (10.0)
10	235 (65.3)	111 (41.4)	124 (34.5)	54 (20.5)	109 (29.9)	48 (17.7)	251 (70.7)	136 (51.9)	189 (52.5)	85 (31.6)	88 (24.5)	30 (11.4)
**Gender**												
Male	339 (47.4)	148 (27.1)	159 (22.1)	66 (12.4)	160 (21.8)	73 (12.6)	361 (50.3)	173 (32.8)	266 (36.7)	113 (20.1)	125 (17.3)	42 (7.9)
Female	375 (53.9)	161 (26.4)	188 (27.4)	71 (11.7)	174 (24.6)	62 (9.9)	318 (46.6)	159 (26.4)	217 (30.8)	91 (14.8)	102 (14.8)	34 (5.6)

* All outcomes significantly lower in 2006 compared to 2002 (*p *≤ 0.01 used due to multiple testing^45^)

^a^43-100 students answers missing per question

^b^ever smoked at least a few puffs of a cigarette

^c^smoked at least one cigarette on at least one day in the last three months

^d^currently smokes at least part of a cigarette in a week

^e^drank at least one alcoholic drink on at least one day in the last three months

^f^drank at least five alcoholic drinks on at least one day in the last four weeks

^g^used marijuana on at least one day in the last three months

Similarly, the proportions of students in each individual school, the proportions of males and females, and the proportions of students in each grade that reported substance use for each of the six outcome measures was significantly lower at follow up than at baseline (Table 4).

## Discussion

This pilot study sought to describe the potential effectiveness and feasibility of a novel comprehensive resilience and protective factor-based intervention on adolescent resilience and substance use. The results suggest that the intervention approach has the potential to decrease the extent of tobacco, alcohol and marijuana use across all students. In addition, the results confirm the feasibility of implementing such an intervention inclusive of a range of explicit adoption strategies within existing school practice. Given the importance of such behaviours to adolescent health, as well as the implications for educational practice in schools, a more rigorous controlled evaluation of the intervention is warranted to confirm the potential suggested by these findings.

Resilience theory was first developed to explain why some disadvantaged children were able to succeed in a context of high personal and environmental risk, whereas other children did not [23,24]. Previous studies have suggested an ability to strengthen the resilience and protective factor characteristics of a number of population groups other than adolescents [64,65]. For example, a controlled trial with college students has reported 5%-10% increases in resilience following a four week intervention [65]. Similarly, in a non-controlled study implemented in primary schools, significant increases in self-esteem, and school and family connection of 23-38% were reported following a five month resilience-based intervention [64]. The findings also extend those of a non-controlled evaluation of the program that formed the basis of the intervention implemented in this study [41]. In that analysis, significant increases in school connection, autonomy experience and help-seeking among adolescents were reported, but not for self-esteem [41]. Although such studies have suggested an ability to increase student resilience and protective factor scores, the clinical significance of such increases is unknown.

Although statistically significant, only modest improvements were found in resilience and protective factors in this study (1-2 point increase). However such a level of improvement at a group or population level may be important from a public health perspective [66]. Further research is recommended of the effect of a resilience intervention such as that described in this study. Similarly, research focused on the standardisation of the resilience measure in an Australian high school population is also recommended.

Given the limited evidence regarding the efficacy of school-based interventions in reducing tobacco, alcohol and marijuana use, the observed differences in prevalence for all six substance use measures in this study are promising. Although the ability to compare study findings is limited due to methodological differences between studies, the observed differences in this study appear larger than the positive effect sizes in previous studies [34,38-42]. For example, in a controlled trial of a protective factor intervention designed to reduce substance use in a cohort of Grade 6 students in the USA, 2% absolute reductions in tobacco (intervention 28% verses control 30%) and marijuana use (intervention 8% verses control 10%) were observed at 3 year follow up [42]. Similarly, in a five and a half year follow up of a randomised controlled trial comparing the effects of two family and school interventions on tobacco, alcohol and marijuana use, relative reductions of 12-21% were reported in the prevalence of smoking initiation and 23% for marijuana initiation [38]. The 47-51% and 58% relative differences found in this study compare favourably to such previous study outcomes.

Similarly, despite the normal developmental trajectories of substance use where prevalence of use increases with age [9,11-13], and the variable intervention exposure across grades, positive substance use results were achieved in this study across all grades, schools and both genders for all outcome measures. These results contrast the inconsistent group effects found in previous resilience focused studies [34,38-42]. For example, as discussed previously, a controlled protective factor intervention in 26 Australian high schools was able to demonstrate decreases in either smoking or marijuana use at follow up, but not for others [39,40]. Similarly, a non-controlled resilience and protective factor intervention in 15 Australian high schools was able to demonstrate decreases in smoking, alcohol or marijuana use in only a limited number of grade and gender groups examined [41].

Whilst consistent decreases in health risk behaviours were observed across all schools, the same was not evident in resilience and protective scores with one school's median score remaining unchanged (school c) despite implementing a similar number of intervention strategies. Future studies, inclusive of measures of implementation fidelity, are required to better determine the association between resilience and substance use, including the changes in both and developmental influences.

The extent to which the various elements of the intervention (for example, the explicit inclusion in this study of strategies that addressed both resilience and protective factors, the use of a health promoting schools approach [22] and the inclusion of strategies to enhance intervention adoption) or the variable intervention dose across schools contributed to the observed outcomes that other studies have not been able to demonstrate is unknown. Further research to determine the differential contribution of such factors on 'school culture' [22], resilience and protective factors, substance use and the association between such outcomes would be of benefit.

Interpretation of the study results should be viewed in light of a number of its characteristics. First, the non-controlled study design and the use of cross sectional data preclude the drawing of causal links between the intervention and the observed outcomes. Although the design does not allow for such attribution, comparison with data from regularly conducted state-wide secondary school surveys suggest that the differences in substance use observed in this study exceed a general declining trend in use across New South Wales [11]. Based on such survey data, the absolute proportion of all 12 to 16 year old students in the state, and all such students, who reported 'ever smoking' decreased by 7% (39% to 32% for both populations) between 2002 and 2005 [11], compared to the 24% absolute difference between 2002 and 2006 observed in this study. The finding that the observed differences in substance use exceeded temporal trends at the state level strengthens the possibility that they may be attributable to the intervention.

Similarly, due to the study design it is unknown whether characteristics of the participating schools or students had an impact on the observed results. It is possible that the greater effect found in this study is due to the particularly low level of disadvantage in the community in which the schools were located. Alternatively, it has been argued that modifying health risks among disadvantaged populations is more difficult, as evidenced by their greater prevalence of health risk behaviours [10]. However the extent to which the level of disadvantage contributed to the effect sizes found is unknown.

Similarly, whilst data suggests that a proportion of students change schools each year across the state [67], the extent to which the rates of such movement occurred in the study schools is not known. During the study period the number of students increased in two of the three schools. Whilst students leaving the school during the study period would not be expected to have an impact on the study outcomes, the entry of new students to the schools during the study period has the potential to have had an impact due to reduced exposure to the intervention. As the effect of this would be to diminish the effect size of the intervention, the reported results could be considered to be a conservative estimate of effect.

Second, whilst the consent rates achieved in this study are typical for school-based research using active consent [68], the risk of non-response bias has been suggested to increase substantially once participation rates fall below 80% [69]. Previous studies have reported non-responding children to have a higher prevalence of health risk behaviours [70], whilst others report inconsistent or no differences in health risk behaviour prevalence [71]. If such an effect occurred, the potential exists that the lower the response rate at follow up may have contributed to the reduction in substance use however the extent to which this may have influenced the findings is unclear.

Third, the small number of participating schools limits the generalizabilty of the results to the broader population of schools. Additionally, the three participating schools are located within one community and the extent to which these results could be generalised to other disadvantaged schools or the broader population of schools is unknown. Future research is required that assesses the efficacy of the intervention in both the general population and high risk populations. Similarly, future studies should include the collection of data regarding the ethnicity of students in order to examine any differential intervention effects for students of different cultural backgrounds.

Finally, as the study relied on adolescent self report of health risk behaviours, the validity of the outcome measures is unknown [72]. Whilst a number of studies have reported that adolescent self report of tobacco use corresponds well with biochemical markers of tobacco smoking [73], options to increase the accuracy of self report exist. The bogus pipeline approach [74] and other methods of data collection, such as web based surveys, have been suggested to have higher participation rates and to increase the reporting of substance use [75,76].

## Conclusions

Despite these limitations, the results of this study confirm the feasibility, and suggest the potential, of a resilience based intervention approach with the inclusion of explicit adoption strategies, in reducing the unacceptably high tobacco, alcohol and marijuana use among adolescents. To further investigate the potential of this approach, future research employing a more rigorous controlled research design across a larger range of schools is required. In the event that such rigorous research confirms this potential, subsequent studies seeking to establish the relative effectiveness and cost effectiveness of the intervention elements is warranted.

## Competing interests

The authors declare that they have no competing interests.

## Authors' contributions

RKH: Performed the data analysis and drafted the manuscript. JD: Participated in the design and coordination of the study, and the critical revision of the manuscript. MF: Participated in the implementation of the intervention, helped in the drafting and participated in the critical revision of the manuscript. JB: Participated in the interpretation of the data and the critical revision of the manuscript. TH: Participated in the acquisition, analysis and interpretation of data and the critical revision of the manuscript. JW: Conceived of the study, participated in its design and coordination, helped draft the manuscript and participated in the critical revision. All authors read and approved the final manuscript.

## Pre-publication history

The pre-publication history for this paper can be accessed here:

http://www.biomedcentral.com/1471-2458/11/722/prepub
